# Chondroprotective Effects of Grapefruit (*Citrus paradisi* Macfad.) Juice in a Complete Freund’s Adjuvant Rat Model of Knee Osteoarthritis

**DOI:** 10.3390/nu15040798

**Published:** 2023-02-04

**Authors:** Reem S. Alazragi, Hanadi M. Baeissa

**Affiliations:** Department of Biochemistry, College of Science, University of Jeddah, P.O. Box 34, Jeddah 21959, Saudi Arabia

**Keywords:** grapefruit juice, knee osteoarthritis, complete Freund’s adjuvant

## Abstract

Osteoarthritis (OA) is a common disorder that can affect any joint in the human body. This study aimed to examine the anti-arthritic properties of high and low doses of grapefruit juice (GFJ), as grapefruit appears to contain anti-inflammatory biochemicals. Forty male Sprague–Dawley rats weighing 170–180 g were divided into five groups. These groups comprised the untreated control group and osteoarthritic (Osteo) rats administered intra-articular injections of Freund’s complete adjuvant (CFA; 0.5 mL; 1 mg/mL) as follows: OA rats administered low doses of GFJ (Osteo+GFJ (low); 5 mL/kg body weight (BW)); OA rats administered high doses of GFJ (Osteo+GFJ (high); 27 mL/kg BW); and OA rats administered diclofenac sodium (Osteo+Diclo) as a reference drug. Injections of CFA induced OA, as indicated by a significant increase in the serum levels of the inflammatory biomarkers C-reactive protein (CRP), interleukin-1β (IL-1β), and (prostaglandin (PGE2), as well as matrix metalloproteinases (MMP-1) and cathepsin K. The synovial levels of glycosaminoglycans (GAGs), tumor necrosis factor (TNF-α), and interleukin 6 (IL-6) also increased, with a concomitant reduction in osteocalcin levels. The administration of either high or low doses of GFJ reduced CRP, IL-1β, PGE2, MMP-1, cathepsin K, and osteocalcin while increasing the synovial levels of GAGs, TNF-α, and IL-6, slowing cartilage degradation and boosting joint function. The results showed comparable histopathological and biochemical responses. A comparison of the treatments showed that high-dose GFJ had a greater chondroprotective effect than low-dose GFJ.

## 1. Introduction

Osteoarthritis (OA) has many clinical symptoms, and the world’s aging population has resulted in OA being an increasingly common problem worldwide. OA causes chronic disability in the spine and in the knee, hip, and hand joints due to its effects on cartilage, synovial membranes, and bone structure [1]. In the later stages, it incrementally degrades cartilage, erodes calcified materials, and compromises the extracellular constituents of the matrix, thereby meaningfully affecting the resistance of the cartilage and its tensile strength. The etiology of OA varies and can include metabolic and genetic and factors, aging, joint damage, obesity, or surgery [2].

Further OA effects are exerted by chondrocytes, which secrete proinflammatory cytokines that trigger synovial tissue loss, causing inflammation and joint deterioration [3]. Chondrocytes then increase matrix biogenesis and secrete anti-inflammatory cytokines to counterbalance the degradative effects of the inflammatory cascade, but biochemical changes in the articular cartilage stop this rise in synthesis by further increasing the degradation of the cartilage. Greater synthetic activity is initially limited to the deep layers of cartilage, causing an imbalance that leads to the degradation of the upper layer. Eventually, chondrocytes may die, thereby hastening the development of OA [4]. Globally, OA affects about 10–17% of the population in Europe, 12–21% in North America, 2–4% in South America, and 16–29% in Asia, Africa, and Middle Eastern countries [5]. Knee osteoarthritis (KOA) is the main cause of chronic disability. The principal clinical symptoms of KOA are joint pain, activity restriction, stiffness, and difficulty in walking [6]. Pathologically, this condition is characterized by cartilage degeneration, subchondral bone matrix damage, and synovitis. One new option for treating OA involves treating the symptoms with anti-inflammatory drugs to provide pain relief. The most common treatments are NSAIDs (nonsteroidal anti-inflammatory drugs) and specific COX-2 (cyclooxygenase-2) inhibitors. However, these medications lead to gastrointestinal complications and can induce heart and mental disorders when taken orally [7]. NSAIDs, in particular, have severe side effects, including hepatotoxicity, which can be asymptomatic or manifest as moderate or reversible increases in hepatic function indices, and can also lead to lethal hepatitis, nephrotoxicity, jaundice, and gastrointestinal disruption [8].

Diclofenac sodium is a popular NSAID-based medication that also has antibacterial, analgesic, antipyretic, and anti-inflammatory attributes [9]. The anti-inflammatory properties of diclofenac sodium are exerted by inhibiting cyclooxygenase (COX) isoenzymes, which decrease prostaglandin biosynthesis. The long-term use of diclofenac sodium can have severe side effects, including mucosal gastric ulcers and platelet disorders, as well as harmful impacts on brain and heart function [10]. The limitations of these new therapies and drugs mean that it is crucial to discover natural products with lower levels of toxicity for the treatment of chronic diseases such as OA; this is the basis for the present study, which examines grapefruit juice’s (GFJ’s) protective action against OA.

Fruit juice beverages are formed by pressing or crushing fresh fruit and are regularly consumed for their apparent health benefits. Bitterness is a problem in some fruit juices; in GFJ it is caused by naringin (4′,5,7-trihydroxyflavanone-7-rhamnoglucoside). Grapefruit (*Citrus paradisi* Macfad., from the Rutaceae family) mostly grows in subtropical climates. It contains nutritional carbohydrates, lipids, proteins, vitamins, and minerals, as well as bioactive compounds including flavonoids, carotenoids, coumarins, and organic acids (e.g., hydroxycinnamic acid). It is also a good source of minerals (K, Na, Ca), vitamins (C, A, E, and B complex), and fiber folate. It is rich in phytochemicals, including limonoids and lycopene [11], which have various health benefits, including anti-inflammatory, anticancer, and weight-loss activities. Various GFJ constituents can also inhibit the DNA damage caused by xenobiotics, as shown in different experimental models [12]. The naringin found in GFJ also has several pharmacological properties, including antioxidant, blood-lipid-lowering, and anticarcinogenic activities, and it inhibits the action of some cytochrome P450 enzymes [13]. Consequently, the purpose of the current study was to estimate the potential beneficial effects of dietary supplementation with GFJ in an experimental rat model of complete Freund’s adjuvant (CFA)-induced OA and to assess the changes in biochemical and histological parameters in this rat model to validate the medicinal use of GFJ for treating or relieving the severity of OA.

## 2. Materials and Methods

### 2.1. Materials

Grapefruits were procured from local markets in Jeddah, Saudi Arabia. CFA (1 mg/mL, parenteral) was acquired from Sigma (St. Louis, MO, USA). Diclofenac sodium (Voltaren^®^ SR 100 mg) was obtained from local markets in Jeddah, Saudi Arabia.

### 2.2. Animals and Treatments

Male Sprague–Dawley rats (adult, *n* = 40; weighing 170–180 g) were used for this research. All rats were kept in an air-conditioned room (25 ± 2 °C) at relative humidity (50 ± 10%) with a 12-h light/dark cycle. The animals were fed ad libitum with a commercial standard diet as recommended, with free access to water. After one week of acclimatization, the rats were randomly separated into the following 5 groups (6 animals/group):

Group 1: animals were intraarticularly injected once into the left knee joint with 0.05 mL of saline (negative control group) [14].

Group 2: rats were anesthetized and received an intraarticular injection of 0.5 mL (1 mg/mL) commercially formulated CFA into the synovial cavity of the right knee; this was the induced positive control group (positive control group) [15].

Group 3: osteoarthritis + low dose GFJ (OA+GFJ). The OA rats were given GFJ at 5 mL/kg body weight (BW) daily for 28 days [16].

Group 4: osteoarthritis + high dose GFJ (OA+GFJ). The OA rats were given GFJ at 27 mL/kg BW daily for 28 days [17].

Group 5: osteoarthritis + diclofenac sodium (Osteo+Diclo). The OA rats were orally administered diclofenac sodium at 10 mg/kg BW in 0.5% dimethyl sulfoxide [18].

All efforts were made to handle the rats humanely and adhere to ethical rules during the experiment.

### 2.3. Sample Collection

With the use of an accurate balance and a manual Vernier caliper, fluctuations in respective body weight and knee diameter during the experimental period were measured. At the end of the research period, all rats were starved overnight and sacrificed under ether anesthesia. The synovial fluid (SYF) was withdrawn with a syringe based on the protocol outlined in [19]. The collected SYF samples were centrifuged at 13,000 rpm/min for 10 min to collect the supernatant for biochemical assays of SYF. The blood samples were obtained by retro-orbital puncture and the serum was detached by permitting blood samples to clot at a temperature of 25 °C for 30 min. The samples underwent centrifugation at 3000 r/pm for 20 min. The serum was stored at −20 °C pending the biochemical analysis.

### 2.4. Biochemical Assay of SYF and Serum

BioVision (Waltham, MA, USA) provided the ELISA kits for assessing IL-6 (interleukin 6; K4145-100) and TNF-α (tumor necrosis factor, ab285327) with sensitivity values of 1 and 5 pg/mL, respectively [20]. The levels of glycosaminoglycans (GAGs; ab289842) in SYF were detected using commercial colorimetric kits provided by BioVision (Waltham, MA, USA) according to the method outlined in [21]. The serum levels of C-reactive protein (CRP), osteocalcin, (matrix metalloproteinases) MMP-1, and cathepsin K were estimated using a specialized sandwich ELISA kit (RayBiotech, USA) according to the manufacturer’s instructions. Prostaglandin (PGE2) and interleukin-1β (IL-1β) were measured in rat serum using a quantitative competitive ELISA kit (Cusabio, Wuhan, China) according to the manufacturer’s guidelines.

### 2.5. Histopathological Examination

For the H&E assay, small specimens from the right knee joints were identified and cut. The samples were fixed in phosphate-buffered formalin (10%, Sigma Aldrich, USA) and decalcified. Samples were splashed with tap water and managed for paraffin blocks. Then, 5 μm thick pieces were cut and subjected to hematoxylin and eosin (H&E) staining [22]. With the use of standard methods, histological samples were prepared for light microscopy (40×) examination.

### 2.6. Statistical Analysis

The results were subjected to one-way analysis of variance (ANOVA) using SPSS software 20.0(SPSS Inc., Chicago, IL, USA). The presented results are the mean ± SEM. A *p* value of < 0.05 was considered significant.

## 3. Results

### 3.1. Effects on Body Weight and Knee Diameter

The data in Table 1 show that injections of CFA produced symptoms of KOA, as evidenced by the increased knee diameter. A significant (*p* < 0.05) decrease in body weight was also clearly detected in the OA rats compared to the control group. The oral administration of GFJ, at either low or high doses, or of diclofenac, significantly (*p* < 0.05) attenuated the increase in knee diameter. A comparison of the treatments showed that a high dose of GFJ was more effective than a low dose. GFJ administration also decreased body weight compared to the untreated osteoarthritic rat group. The weight gain was lower after the administration of high doses of GFJ.

### 3.2. Effects on serum inflammatory biomarkers

Table 2 shows that CFA injections caused knee inflammation, which can be explained by the significant (*p* < 0.05) increase in the levels of inflammatory biomarkers (CRP, IL-1β, and PGE2). The administration of GFJ at low or high doses significantly suppressed this increase. The efficacy was better for high-dose GFJ than for low-dose GFJ and resembled that of diclofenac.

### 3.3. Effects on serum MMP-1, cathepsin K, and osteocalcin

Table 3 shows that CFA injections induced a significant (*p* < 0.05) increase in MMP-1 and cathepsin K serum levels, with a significant concomitant reduction in osteocalcin compared to the control rat group. The oral administration of GFJ at either low or high doses, or treatment with diclofenac, lowered these levels significantly (*p* < 0.05). High-dose GFJ was more effective than low-dose GFJ.

### 3.4. Effects on GAG, TNF-α, and IL-6 in SYF

**Figure 1:**
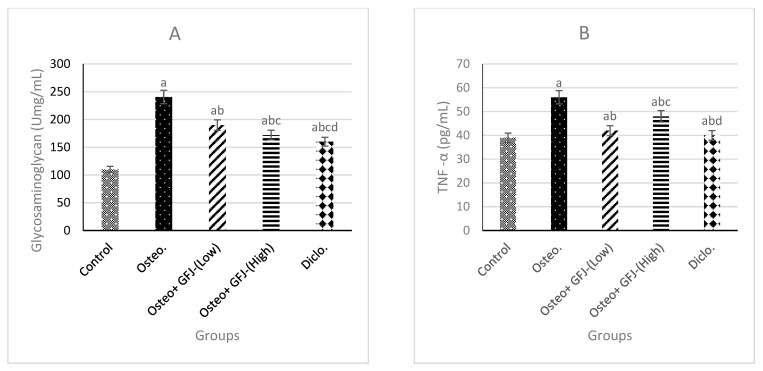

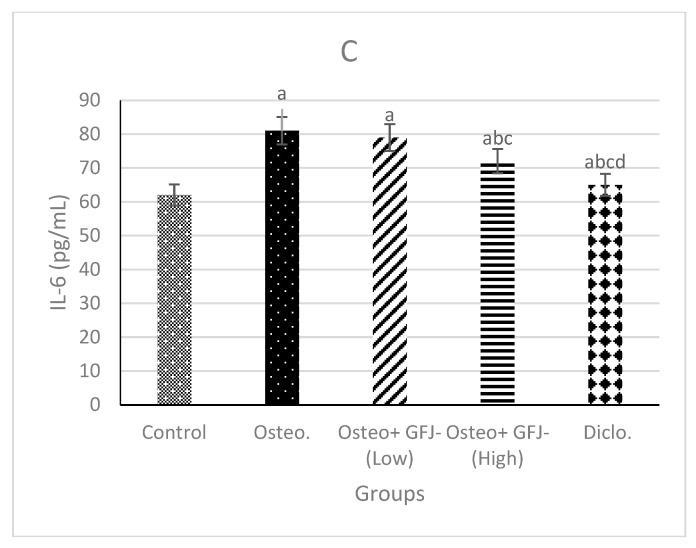
The influence of various treatments on synovial fluid levels of glycosaminoglycan (**A**), TNF-α **(B**), and IL-6 (**C**). Osteo = OA, GFJ = grapefruit juice, diclofenac = sodium diclofenac group. a = significant versus the control group; b = significant versus the osteoarthritic group; c = significant versus the Osteo+GFJ (low) group; d = significant versus the Osteo+GFJ (high) group.

Figure 1 displays the effects of different treatments on the synovial fluid levels of GAG, TNF-α, and IL-6 (Figure 1A–C, respectively). CFA injections induced a significant (*p* < 0.05) increase in the synovial levels of GAG, TNF-α, and IL-6 relative to the control. Moreover, the oral administration of GFJ at low or high doses and treatment with diclofenac lowered these levels compared to the osteoarthritic rat group.

### 3.5. Effects on the macroscopic apperance of the articular cartilage

**Figure 2:**
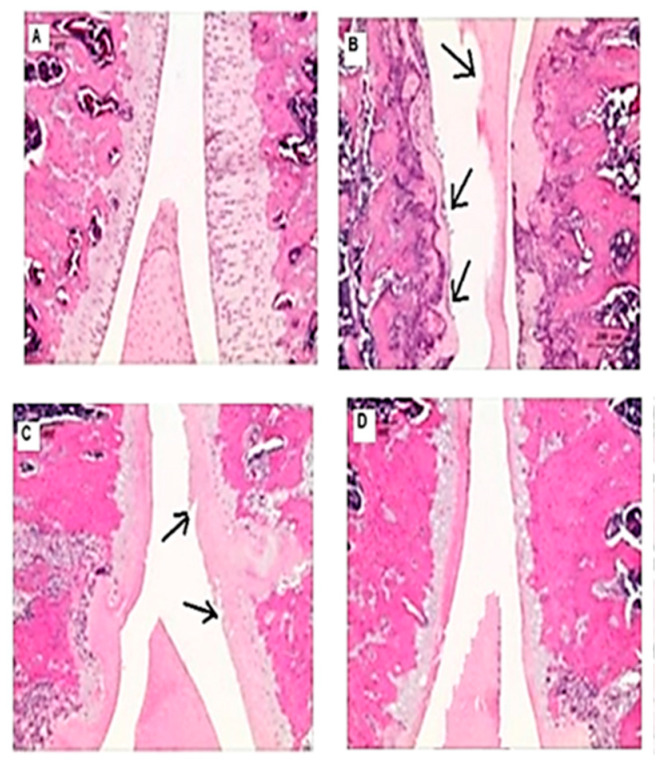

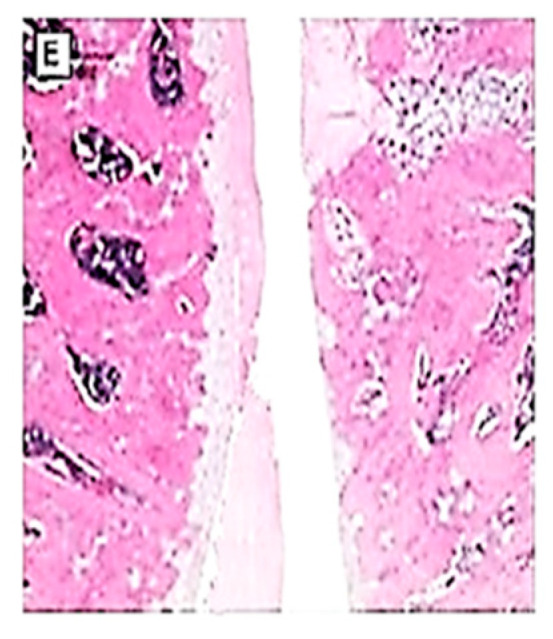
Micrograph of histological changes in the articular cartilage of rat knees. (**A**): the knee of a normal control rat, showing articular cartilage with a smooth surface and normal cellularity (H&E × 100). (**B**): the knee of an osteoarthritic rat, showing reduced cartilage thickness and an abnormal matrix (black arrows); the surface is fibrillated and irregular. (**C**): the knee of an Osteo+GFJ (low) rat, showing an abnormal matrix but a slight improvement in the surface (black arrows). (**D**): the knee of an Osteo+GFJ (high) rat, showing an improvement in cartilage thickness, a reduction in the surface fibrillation, and the restoration of a normal surface appearance. (**E**) the knee of an Osteo+Diclo rat, showing recovery in cartilage thickness and the restoration of the normal cartilage surface (H&E × 200).

Articular cartilage sections from the control group showed normal surface tissue (Figure 2A). On the other hand, the osteoarthritic group showed reduced cartilage thickness and an abnormal matrix (Figure 2B). Comparing treatment groups, the Osteo+GFJ (low) group showed an abnormal matrix with a slight improvement in the surface (Figure 2C). Whereas, the Osteo+GFJ (high) group showed a manifest improvement in the cartilage thickness and a restoration of the normal surface appearance (Figure 2D). Figure 2E showed a recovery in the cartilage thickness and a restoration of the normal cartilage surface.

## 4. Discussion

OA is a health concern for aging people and leads to joint pain and chronic disability. OA affects 50% of people aged older than 65 years and current statistics indicate that it will overtake diabetes as the leading cause of disability by 2040 [23]. Deterioration of the articular cartilage and irregular remodeling of the subchondral bone are common pathological changes in OA. The subchondral bone and articular cartilage work together to keep the joint’s internal environment balanced and stable [24]. Diclofenac sodium is a standard drug used to treat OA and is the most commonly used reference drug for laboratory research and the preclinical development of novel antiarthritic agents [25]. In this study, we investigated the potential of dietary supplementation with GFJ in an investigational rat model of CFA-induced OA. Complete Freund’s adjuvant-caused arthritis is a typical chronic test model and is linked to an immune-mediated inflammatory reaction, oedema, and soft tissue thickening [26]. The penetration of diclofenac, administered as a diclofenac sodium topical solution, is enhanced by dimethyl sulfoxide [10]. Whether administered orally or topically, diclofenac penetrates the synovial fluid and enters systemic circulation in OA patients. However, the elimination half-life of synovial fluid is three times longer than that of plasma, suggesting that it has a more sustained therapeutic effect [27]. The results of the present study showed a weight reduction in the OA rat group. Reductions in body weight during inflammation result from the poor absorption of nutrients through the intestinal wall [28]. Extensive use of diclofenac sodium as an anti-inflammatory drug normalizes the absorption process, restoring body weight. In the case of OA, diclofenac improves the intestinal absorption of nutrients while reducing the distress triggered by acute OA [8].

Treatment with GFJ at either high or low doses also counteracted OA symptoms, with better effects observed at high doses. The effects of GFJ can be attributed to GFJ’s high naringin content. Naringin is the major flavonoid component in GFJ, as reported in numerus studies [29,30]. It is a safe bioactive compound. Its toxicity was investigated in Sprague–Dawley rats for 6 months. Naringin was given to rats by oral routs for 1, 32, 93, and 184 days at a doses of 25, 250, and 1250 mg/kg/day; no death or toxic effects were documented [31].

Naringin has broad biological effects on human health. It decreases lipid peroxidation biomarkers and protein carbonylation, promotes carbohydrate metabolism, increases antioxidant defenses, scavenges reactive oxygen species, modulates immune system activity, and exerts anti-atherogenic and anti-inflammatory effects [32]. Naringin effectively lowers blood glucose and cholesterol concentrations and improves insulin signaling [33]. For instance, three weeks of treatment of overweight patients with GFJ three times a day induced significantly reduced amounts of total cholesterol and low-density lipoproteins [34]. Another study revealed that GFJ decreased plasma triglyceride levels in a dose-dependent manner [35], which can be attributed to the capacity of citrus flavonoids to constrain apolipoprotein B secretion from hepatocytes, thus reducing body weight.

Our investigation showed that CFA-induced arthritis was characterized by a significantly enhanced general inflammatory reaction and increases in the synthesis of cytokines, such as interleukin-β1, thereby triggering PGE-2 in the inflammatory response and causing cartilage injury and degradation. As noted in [36], these proinflammatory cytokines are present as indispensable intermediaries in the initial stages and during the development of OA. Inflammation involves several processes, such as inflammatory cell activation, proinflammatory cytokine production, and the release of inflammatory intermediaries, such as PGE2, causing symptoms of inflammation, including swelling, fever, redness, and pain [37]. Studies suggest that pro-inflammatory cytokines, such as IL-1β and IL-6, formed by a cytokine response, may contribute to degenerative processes in the joints and accelerate the degradation of the cartilage matrix. For instance, IL-1β can lead to dramatic increases in the matrix MMP expression and encourage the development of OA [38]. The results of the present study are in agreement with a previous report that increased serum levels of IL-1β are a characteristic feature of OA [39].

CFA injections triggered a substantial increase in the serum levels of CRP relative to the healthy group, following earlier findings that CRP can function as an indicator for the prediction and diagnosis of OA [40]. Previously, researchers proposed that CRP estimations relating to OA probably reflect a complex interaction of joint inflammation and systemic inflammation [41]. Moreover, inflamed joints are likely to enhance CRP synthesis via the liver by cytokines originating from joint tissue. This hypothesis is supported by the most recent KOA study [42]. In the current investigation, CRP levels dramatically improved in animals treated with diclofenac, in agreement with earlier findings [43]. Diclofenac is a potent inhibitor of prostaglandin PGE2 formation, reducing inflammation and arthritic pain.

Treatment with GFJ at either high or low doses showed similar results to diclofenac; this result is consistent with that of a previous study that showed improvements in inflammatory markers in obese rats treated with grapefruit extract [44]. Similar to other citrus fruits, grapefruit is high in the flavonoid compounds naringenin/naringin and hesperidin, which have anti-tumor, antioxidant, anti-inflammatory, and antidiabetic properties [45,46]. Other reports have shown the possible benefits of naringin treatment in reducing ultraviolet-induced inflammation in rat skin by reducing interleukin levels [47]. Naringin and hesperidin appear to modulate many growth factors and pro-inflammatory responses, but most research has concentrated on these flavonoids’ antioxidant and antimicrobial effects [48]. Narirutin (a flavanone in grapefruit extract) has been studied for its inflammation-reducing effects, but minimal evidence supports its efficacy [48,49]. GFJ was found to decrease gastric lesions and diarrhea in a rat colitis model and to increase the glutathione content, probably as a result of the antioxidant and anti-inflammatory properties of GFJ [50]. Previous work on the effects of GFJ in people with rheumatoid arthritis and other inflammatory disorders showed that eating grapefruit daily helped to alleviate their symptoms due to the presence of chemicals that block prostaglandins, such as PGE2 [33].

The findings of the existing trial showed that CFA injections increased the serum levels of MMP-1 and cathepsin K, with a significant concomitant reduction in osteocalcin; these findings concur with other reports of high expressions of MMP-1 and cathepsin K in arthritis [51]. These biomarkers have also been shown to play significant roles in collagen degradation [51]. Other studies have shown increased MMP-1 and cathepsin K expression in arthritis [52]. Treatment with CFA also significantly reduced osteocalcin levels in the rat OA model. Osteocalcin is a protein secreted exclusively by osteoblasts and is assumed to play a significant part in metabolic control in the body. Since osteoblasts generate osteocalcin, they are commonly considered a marker of bone formation, and serum osteocalcin is thought to indicate osteoblastic activity and bone remodeling [53]. In arthritis, osteoblastic function is suppressed, while osteoclastic activity is promoted, resulting in a decrease in serum osteocalcin levels. These levels clearly improved in response to GRJ treatment at both high and low doses, in agreement with previous findings of an improvement in bone quality with the administration of orange juice and GFJ [54]. Similarly, hesperidin also restricted ovariectomy-induced bone damage in rats [55]. Likewise, as reported by [56], limonoids, a group of oxygenated terpenoids in GFJ, have been shown to have antioxidant activity and may shield bones from recourse. GFJ is also rich in flavonoids, limonoids, and vitamin C; these compounds may shield bones. Naringin enhances osteocalcin expression and effectively reflects ovariectomy-induced osteoporosis in rats, suggesting that naringin management may constitute an efficient remedy for osteoporosis [57]. Likewise, administering 40 mg/kg BW of naringin to Wister osteoarthritic rats reduced pain, improved tissue morphology, and inhibited MMP-3 expression and NF-κB pathway signaling [58]. Several phytogenic compounds have been considered potential suppressors of MMPs and these can be further treated to develop drugs suitable for treating rheumatoid arthritis and inflammation [59]. Collectively, the available studies support the role of grapefruit flavonoids in inhibiting MMP-1 expression.

The evaluation of the connection between structural injury and synovial inflammation during the development of OA suggests that inflammatory mediators may indicate the progression of OA in patients [52]. Several trials on OA have confirmed that inflammatory mediators play a significant role in the progression and expansion of cartilage destruction [60]. Our findings revealed a considerable increase in the GAG, TNF-α, and IL-6 levels in the synovial fluid of osteoarthritic animals compared to their healthy counterparts. GAG is a key constituent of joint fluid cartilage and soft connective tissues [21,61]. During the degradation and inflammation of cartilage, large quantities of GAG are released from the degrading cartilage matrix and can be detected in blood serum or SYF [21]. Therefore, GAGs can serve as clinical markers for OA and indicate loss of the load-bearing capacity of the cartilage [62]. The increase in GAG released into the synovial fluid varies according to the grade of OA [42]. Other important inflammatory molecules, such TNF-α and IL-6, are involved in the progression of OA. High levels of IL-6 have been reported in the synchronous fluids of patients with focal cartilage defects [63] and individuals with high levels of circulating IL-6 may be more susceptible to radiation knee infection [64]. One investigation of a canine model of OA [65] showed that high levels of TNF-α and IL-6 in synovial fluid were related to early OA—findings that are consistent with our own. The results of the present work concur with earlier data on flavonoid effects on GAG release and with the suggestion that flavonoids such as neohesperidin, naringin, and neoeriocitrin are potential therapeutic agents for protecting cartilage tissue [63,66]. Hesperidin exhibited substantial anti-inflammatory activities in vitro in a macrophage cell line and in vivo in an acute liver injury model, reducing the levels of IL-6 and TNF-α [67,68]

Other GFJ components, such as quercetin and kaempferol, have been identified as inhibitors with protective effects against inflammatory bowel disease [32]. The histological results in the present study confirmed the presence of blood biochemistry alterations, as OA-diminished cartilage thickness and the formation of an irregular fibrillated cartilage surface and an abnormal matrix intensity (Figure 2). Changes in synovial tissues and bone metabolism can occur in OA [45], along with the destruction of cartilage [69]. Moreover, OA is pathologically characterized by fluctuations in the degradation of cartilage, changes in the subchondral bone matrix, and the induction of synovial inflammation [38]. Furthermore, increased subchondral injury, the thinning of the trabecular system, and the degradation of the cartilage system lead to deteriorations in knee synovial tissue rather than normal development [43].

## 5. Conclusions

This study demonstrated the anti-inflammatory properties of GFJ in a rat OA model. The results indicated the potential of GFJ in the treatment of OA by demonstrating its ability to protect different articular tissues, especially joint tissues, against inflammation. The present work showed that treatment with GFJ at either high or low doses showed similar results to those obtained with diclofenac, improving the levels of inflammatory markers. However, the anti-inflammatory properties were more pronounced with a high dose than with a low dose of GFJ. The administration of GFJ significantly suppressed the levels of CRP, IL-1β, and PGE2. Moreover, GFJ administration lowered the levels GAG, TNF-α, and IL-6 relative to the control group. These improvements resulted from the antioxidant and anti-inflammatory properties of GFJ, as it contains flavonoids such as naringin and hesperidin [45,46]. These findings suggest that GFJ may have therapeutic potential in the treatment of OA.

## Figures and Tables

**Table 1:** Effect of grapefruit juice (GFJ) on body weight gain and knee diameter in an OA rat model.

Groups	Body Weight Gain (g)	Knee Diameter (mm)
Control	84.4 ± 2.47	1.6
Osteo.	31.5 ± 3.28 ^a^	2.9 ^a^
Osteo+GFJ (low)	52.5 ± 1.66 ^ab^	2.5 ^ab^
Osteo+GFJ (high)	39.6 ± 2.63 ^abc^	2.2 ^abc^
Osteo+Diclo	58.7 ± 2.01 ^abcd^	2.0 ^abcd^

^a^ Significant versus the control group; ^b^ significant versus the OA group; ^c^ significant versus the Osteo+GFJ (low) group; ^d^ significant versus the Osteo+GFJ (high) group.

**Table 2:** Effect of grapefruit juice (GFJ) on the serum levels of CRP, IL-1β, and PGE2 in an OA rat model.

Groups	CRP (pg/mL)	IL-1β (pg/mL)	PGE2 (pg/mL)
Control	20.60 ± 1.44	71.87 ± 1.95	329.78 ± 8.3
Osteo.	42.65 ± 3.28 ^a^	267.44 ± 3.68 ^a^	789.66 ± 11.6 ^a^
Osteo+GFJ (low)	32.54 ± 3.65 ^ab^	210.92 ± 4.28 ^ab^	537.53 ± 15.2 ^ab^
Osteo+GFJ (High)	27.76 ± 2.78 ^abc^	170.48 ± 3.68 ^abc^	490.89 ± 11.1 ^abc^
Osteo+Diclo	24.87 ± 2.26 ^abcd^	101.55 ± 4.68 ^abcd^	395.61 ± 12.4 ^abcd^

^a^ Significant versus the control group; ^b^ significant versus the osteoarthritic group; ^c^ significant versus the Osteo+GFJ (low) group; ^d^ significant versus the Osteo+GFJ (high) group.

**Table 3:** Effect of grapefruit juice (GFJ) on serum levels of osteocalcin, MMP-1, and cathepsin K in all experimental groups.

Groups	Osteocalcin (pg/mL)	MMP-1 (pg/mL)	Cathepsin K (pg/mL)
Control	13.60 ± 1.44	76.87 ± 1.95	48.78 ± 8.3
Osteo.	4.55 ± 1.87 ^a^	163.76 ± 8.43 ^a^	142.66 ± 11.6 ^a^
Osteo+GFJ (low)	10.56 ± 1.88 ^ab^	132.85 ± 5.88 ^ab^	114.53 ± 15.2 ^ab^
Osteo+GFJ (high)	7.12 ± 1.83 ^abc^	92.54 ± 3.98 ^abc^	89.89 ± 11.1 ^abc^
Osteo+Diclo	8.87 ± 1.65 ^abcd^	120.64 ± 6.96 ^abcd^	72.61 ± 12.4 ^abcd^

^a^ Significant versus the control group; ^b^ significant versus the osteoarthritic group; ^c^ significant versus the Osteo+GFJ (low) group; ^d^ significant versus the Osteo+GFJ (high) group.

## Data Availability

Not applicable.
